# Assessing the emergence and reliability of cognitive decline over the life span in Fisher 344 rats using the spatial water maze

**DOI:** 10.3389/fnagi.2014.00002

**Published:** 2014-01-21

**Authors:** Michael Guidi, Ashok Kumar, Asha Rani, Thomas C. Foster

**Affiliations:** Department of Neuroscience, McKnight Brain Institute, University of FloridaGainesville, FL, USA

**Keywords:** aging, F344 rats, hippocampus, episodic memory, reference memory, learning and memory, spatial water maze

## Abstract

The spatial water maze is routinely used to investigate hippocampal-dependent spatial memory and the biological mechanisms that underlie variability in cognitive decline during aging. The utility of the task for repeated testing in order to examine the trajectory of cognitive decline and to prescreen animals prior to therapeutic interventions maybe limited due to carryover effects of repeated training. The current study examines the role of carryover effects, as well as the reliability of individual differences, in determining age-related impairment on episodic and reference memory versions of the water maze task. Results indicate that impaired acquisition of episodic spatial information emerges in middle-age and the propensity for impairment increases with advancing age. While learning was variable across animals, acquisition deficits for episodic information were reliable across training sessions in middle-age and aged rats. A significant impairment in the 24~h retention of episodic spatial information was observed in aged animals. When animals were trained to the same location (i.e., reference memory), an impairment was limited to the rate of acquisition in aged animals. However, with continued training, all aged animals were able to acquire a reference memory and no age differences were observed in the 24~h retention of a spatial reference memory. Together, the results point to a progressive impairment in episodic spatial memory with advancing age and suggest that tests of episodic spatial memory are reliable and more sensitive than reference memory for detecting cognitive decline.

## INTRODUCTION

Episodic memory for information that encompasses a specific temporal and spatial context is particularly vulnerable to age-related decline in humans (Sharps and Gollin, 1987; Zelinski and Light, 1988; Cherry and Park, 1993; Uttl and Graf, 1993; Spencer and Raz, 1995; Newman and Kaszniak, 2000; de Jager et al., 2002; Iachini et al., 2005). For animal models of aging, behavioral tasks should be sensitive enough to detect the emergence of memory impairments in middle-age, as well as assess the severity of the impairment, in order to examine the biological mechanisms responsible. Hippocampal-dependent measures that are sensitive to the emergence of memory deficits include contextual memory during fear conditioning, retention of inhibitory/passive avoidance, and spatial memory examined on various mazes including the radial arm maze, T-maze, and water maze (reviewed by Foster et al., 2012). Indeed, episodic spatial memory deficits emerge in middle-age (Jucker et al., 1988; Ando and Ohashi, 1991; Markowska and Savonenko, 2002; Bizon et al., 2009; Kumar and Foster, 2013). With advancing age, this initial deficit may progress toward a more serious cognitive deficit that mimics a hippocampal lesion, including an inability to learn about spatial relationships that remain constant across days of training (i.e., spatial reference memory deficits).

The water maze task can be designed to specifically examine spatial episodic/working memory or reference memory and has been employed extensively to study hippocampal function. The main distinction between which form of spatial memory is taxed depends on whether the escape platform position changes across training sessions (i.e., trial-independent or trial-dependent). Episodic memory training, sometimes denoted as match-to-place, is trial-dependent as the spatial location of the escape platform changes for each day of training, and the animal is required to learn this new escape location. Thus, episodic spatial memory involves the acquisition of novel or flexible spatial information during each training session. In contrast, reference memory is trial-independent, and depends on learning the spatial aspects that remain constant. This includes the location of the escape platform, which remains in the same spatial location across training sessions.

The various versions of the water maze task have been used in cross sectional aging studies in order to showcase the individual differences in spatial memory impairments as well as the progressive nature of the impairments, such that the proportion of impaired animals increases with advancing age (Frick et al., 1995; Lindner, 1997; Shukitt-Hale et al., 1998). However, the utility of the reference memory task for longitudinal studies or repeated testing is limited by carryover effects due to the strength of the acquired spatial mapping strategy. Specifically, once a spatial search strategy has been acquired, this behavior can be very strong due to reinforcement as a result of repeated training to the same spatial location across multiple days. When animals are retested for acquisition of a new spatial reference memory, even after several months, they rapidly acquire a memory for new reference location. In this case, age-related differences that may be evident in a cross sectional study are not observed in longitudinal studies (Dellu et al., 1997; van Groen et al., 2002; Hansalik et al., 2006). Furthermore, when the task is used as a pretreatment screen, the strength of the reference memory mapping strategy may act as a confounding factor, which can reduce the apparent effectiveness of therapeutic interventions (Rowe et al., 2003) or provide protection against treatments to disrupt spatial memory (Buresova et al., 1986; Saucier et al., 1996; Inglis et al., 2013). As such, the carryover effects limit the use of the reference memory version of the task for longitudinal studies to detect the emergence of impairments and progression of cognitive decline (Foster et al., 2012).

Episodic spatial memory may be more amenable to repeated testing for longitudinal studies as impairments tend to increase during aging despite repeated testing (Dellu et al., 1997; Kikusui et al., 1999). In addition, impairments may be more consistent with repeated testing such that within-subject impaired or unimpaired performance is maintained across multiple testing sessions (Sabolek et al., 2004). However, little is known concerning the reliability of episodic spatial memory deficits following repeated testing on the water maze. The current study employed repeated training to the same location across sessions (reference memory) or training to novel locations across sessions (episodic memory) to examine sensitivity to age-related impairment, the reliability of individual differences in performance, and possible carryover influences associated with repeated testing.

## MATERIALS AND METHODS

### ANIMALS

Procedures involving animal subjects have been reviewed and approved by the Institutional Animal Care and Use Committee and were in accordance with guidelines established by the U.S. Public Health Service Policy on Humane Care and Use of Laboratory Animals. Male Fischer 344 rats, young (5–8 months, *n* = 36), middle-age (12–14 months, *n* = 46), and aged (20–22 months, *n* = 57), were obtained from the National Institute on Aging colony at Harlan Laboratories Inc. through the University of Florida Animal Care and Service facility. Animals were maintained on a 12:12 h light/dark cycle, and provided *ad libitum* access to food and water.

### BEHAVIORAL STUDIES

#### Spatial water maze

**FIGURE 1:**
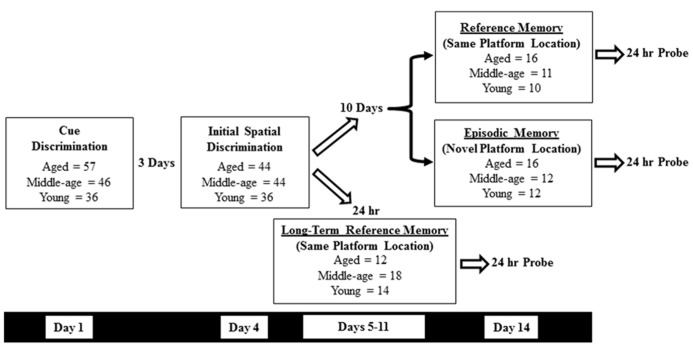
**A schematic overview of the experimental design and training cohorts used in the current study.** Following cue (day 1) and the initial spatial discrimination training (day 4), animals were further divided into three groups for additional water maze testing: one day spatial reference memory (day 14), one day spatial episodic memory (day 14), and long-term spatial reference memory (days 5–11). A retention probe trial was conducted 24 h after the final training trial in each condition.

Methods employed to assess sensory-motor and memory deficits on the water maze have been published previously (Foster et al., 2003; Carter et al., 2009; Kumar et al., 2012; Kumar and Foster, 2013; Speisman et al., 2013a,b). Animals were trained in a black tank, 1.7 m in diameter, positioned in a well-lit room containing (when appropriate) an assortment of two- and three-dimensional cues. Water (27 ± 2°C) was maintained at a level approximately 8 cm below the surface of the tank. Behavioral data was acquired with Noldus EthoVision computer tracking software (Noldus Information Technology, Leesburg, VA, USA) and included cumulative path-length and latency to escape to the platform (12 cm diameter) during training trials. A schematic overview of the experimental design, as well as a breakdown of the animal training groups is provided for in **Figure 1**.

#### Cue discrimination training

Rats were first (day 1) trained on the cue discrimination version of the water escape task. Animals were habituated to the pool by allowing for a 30 s free swim and four-guided attempts to climb onto the escape platform from the four different cardinal directions. The platform was extended approximately 1 cm above the water level and a white Styrofoam flag was attached. Training consisted of five blocks comprised of three trials per block with all training massed into 1 day. For each trial, the platform position and start location was randomized. Inter-trial intervals were 20 s and inter-block intervals were approximately 15 min. For each trial, a rat was placed in the water in one of four equally spaced start locations (N, S, E, and W) and was allowed 60 s to escape onto the platform. If an animal did not escape the water maze within the allotted time, the rat was gently guided to the platform. Rats remained on the platform between trials. After each trial block, rats were placed in their respective home cages under warming fans in order to prevent hypothermia. Rats that failed to find the visible platform at least two times during the last three trials were removed from the study and were not included in the analysis.

#### Initial spatial discrimination training

Three days following cue training (day 4), animals were trained on the spatial discrimination task. For spatial discrimination, the escape platform was hidden approximately 1.5 cm beneath the water level and remained in the same location relative to the distal cues in the room for the duration of the initial spatial training. Training consisted of six blocks of three trials with all training massed into a single day. Inter-trial intervals and inter-block intervals were similar to what was reported for cue training. On each trial, the rat was placed in the water at one of four start locations. The rat had 60 s to escape during each trial; if the animal did not escape within the allotted time, it was again guided to the platform. Rats remained on the platform between trials and in home cages under the heat lamp after each block. Start locations were randomized across each trial. Escape latency and escape path-length was measured. Fifteen minutes following the end of training on block 5, a free-swim probe trial was administered in order to test learning. The probe trial was followed with a refresher-training block (block 6) in order to reinforce the platform location.

For probe trials, the platform was removed and the animal placed in the tank for 60 s. Behavioral measures acquired during the 60 s probe trial included the time spent searching the goal and opposite quadrant. A spatial discrimination index was computed according to the formula (*G* - *O*)/(*G* + *O*) where *G* and *O* represent the percent of time spent in the goal quadrant and quadrant opposite the goal, respectively (Foster et al., 2003; Kumar et al., 2012; Kumar and Foster, 2013; Speisman et al., 2013a,b). Discrimination index scores take into account the quadrant to be approached (the “goal quadrant”) and the quadrant to be avoided (the “opposite quadrant”), and often produce a higher fidelity memory index for aged rats that frequently make wide sweeping turns due to reduced agility (Foster, 2012b; Guidi and Foster, 2012). In addition, the use of a discrimination score permits a measure of the acquisition of a spatial search strategy since random swimming is expected to produce a discrimination index of 0.

#### Reference and episodic memory training

For subsequent spatial training, all external spatial cues remained the same. At this point, animals in each age category were randomly assigned to one of three different groups. One group received long-term (7 days) spatial reference memory training initiated 24 h after the initial spatial training (days 5–11) to the same platform location (see below). For the other two groups, 10 days following the initial spatial discrimination training (day 14), they received a second single day of training for spatial reference memory or spatial episodic memory. For the reference memory group, training was conducted with the escape platform located in same physical location used in the initial spatial discrimination paradigm. For the episodic memory group the platform location was shifted to a new location relative to the distal cues. A single probe trial was performed at a similar time point as previously described; 15 min following the end of training on block 5 with the subsequent refresher block. Retention for platform location was tested 24 h later using another free-swim probe trial.

#### Long-term reference memory training

For reference memory training, most studies employ three to four trials per session and training is conducted over several consecutive days (Foster, 2012b). Due to possible age-related differences in retention of a reference memory over the 10-day period, a third group of animals received spatial reference memory training starting 24 h following the initial spatial discrimination training. The platform remained in the same location as the initial spatial training and the methods remained the same with the exception that only one block (four trials) of training was performed per day for seven consecutive days. On the eighth day, 24 h day following the last day of training, a retention probe was performed to assess memory for the platform location.

### STATISTICAL ANALYSES

All statistical analyses were performed using StatView 5.0 (SAS Institute Inc., NC, USA). Analysis of variance for repeated measures was used to determine significant main effects and interactions across days of training. Fisher’s protected least significant difference *post hoc* comparisons with *p* < 0.05 were employed to determine specific differences. Finally, in order to determine whether each group was employing a spatial search strategy, the discrimination index measures were compared to that expected by chance (i.e., a discrimination index score of 0) using one-group Student’s *t*-tests.

## RESULTS

### CUE DISCRIMINATION TASK

**FIGURE 2:**
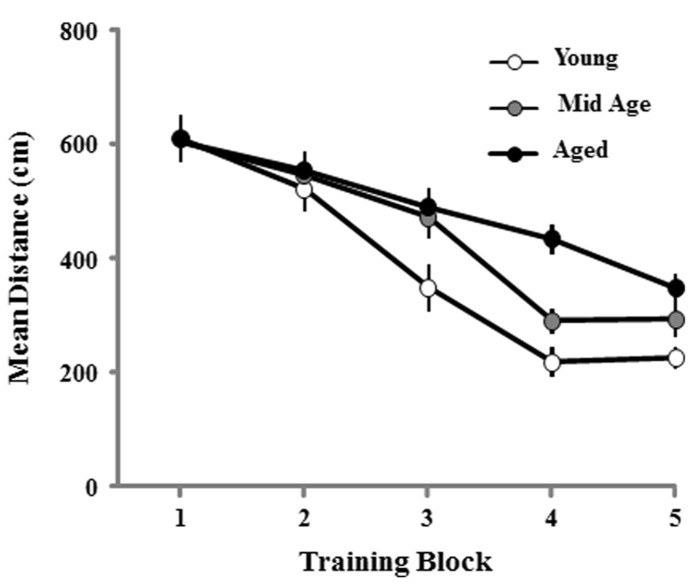
**Mean path length to find the escape platform during the cue discrimination task in the water maze.** Data represents the mean distance traveled (±SEM) per training block in the cue discrimination task for young (open circle, *n* = 36), middle-age (Mid Age, gray circle, *n* = 44), and aged (filled circle, *n* = 44) rats.

The cue discrimination version of the water maze was employed to assess sensory-motor influences on the ability to perform the task and provided animals training on the procedural aspects of the water maze. Thirteen aged and two middle-age animals failed to meet the criterion of the cue task and were removed from the study. For the remaining aged (*n* = 44), middle-age (*n* = 44), and young (*n* = 36) rats, a repeated measure ANOVA on the distance swam to reach the visible escape platform indicated main effects of training [*F*(4,484) = 75.738; *p* < 0.001] and age [*F*(2,121) = 6.809; *p* < 0.01] as well as an age × training interaction [*F*(8,484) = 2.958; *p* < 0.01]. *Post hoc* tests indicated that the young group was different from the other two, with young exhibiting the shortest and aged rats the longest path lengths. However, repeated measures ANOVAs performed for each age group confirmed that each cohort exhibited a decrease in escape path length indicating learning on the task (*p* < 0.0001; **Figure 2)**.

### INITIAL SPATIAL DISCRIMINATION TASK

**FIGURE 3:**
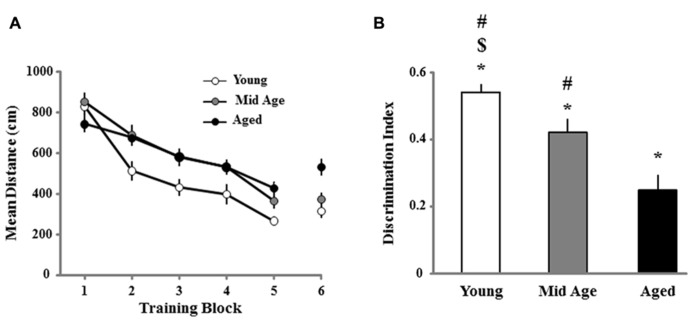
**Animal’s performance on the spatial discrimination task.** **(A)** The mean path length to find the escape platform during the spatial discrimination task in the water maze. Data represents the mean distance traveled (±SEM) per training block in the spatial discrimination task for young (open circle, *n* = 36), middle-age (Mid Age, gray circle, *n* = 44), and aged (filled circle, *n* = 44) rats. **(B)** Mean discrimination index scores (bars + SEM) for the acquisition probe trial, instituted in between blocks 5 and 6. Asterisk indicates significant difference from chance performance, while the dollar and pound signs indicate significant difference from middle-age and aged, respectively.

Three days following the cue discrimination task, all animals were provided a single day of training on the spatial version of the water maze. **Figure 3A** illustrates the decrease in escape path length, organized into blocks of training trials, for young, middle-age, and aged rats. A repeated measures ANOVA indicated a main effect of training [*F*(5,605) = 65.337; *p* < 0.0001] and age [*F*(2,121) = 8.914; *p* < 0.0002]. *Post hoc* tests indicated that young animals were significantly different from both the middle-age and aged cohort, which were not different from each other. Despite these differences, all age groups exhibited learning, as repeated measures ANOVAs performed for each age confirmed an effect of training (*p* < 0.0001; **Figure 3A)**.

A probe trial performed between the fifth and sixth block of the spatial training was employed as a measure of acquisition (**Figure 3B**). One group *t*-tests comparing the discrimination index scores to chance (i.e., a discrimination score = 0) indicated that the scores for each age group were above chance, indicating that all age groups acquired a spatial search strategy. However, an ANOVA on the discrimination index scores indicated an age effect [*F*(2,121) = 13.751; *p* < 0.0001] and *post hoc* analyses indicated that all age groups were significantly different from one another with young and aged rats exhibiting the highest and lowest discrimination index, respectively. The lowest discrimination index score for young animals (score = 0.259) was used as a cut-off criterion for characterizing older animals as learning impaired. The results confirm that aging is associated with an increased proportion of animals classified as learning impaired, which was 23% (10 of 44) for middle-age and 61% (27 of 44) for aged animals. Finally, we determined whether learning on the spatial task was related to performance on the cue task. Regression analysis was performed for the escape path during the last block of cue training and last block of the spatial training within each age group. The results indicate no correlation between the escape path length from the last block of cue training and the final block of spatial training for each age group. Together, the results indicate a progressive impairment in the acquisition of spatial information with advancing age.

### EFFECTS OF REPEATED TESTING: REFERENCE AND EPISODIC MEMORY

At this point, animals in each age category were randomly assigned to three different groups. One group received long-term spatial reference memory training (7 days, four trials/day) to the same platform location (see below). The other two groups received a second single day of spatial discrimination training starting 10 days following the first behavioral assessment. For those rats reassessed 10 days later, one subset received reference memory training to the same platform position (reference: young, *n* = 10; middle-age, *n* = 11; aged, *n* = 16) while a second cohort received episodic memory training using a novel platform position (episodic: young, *n* = 12; middle-age, *n* = 12; aged, *n* = 16).

**FIGURE 4:**
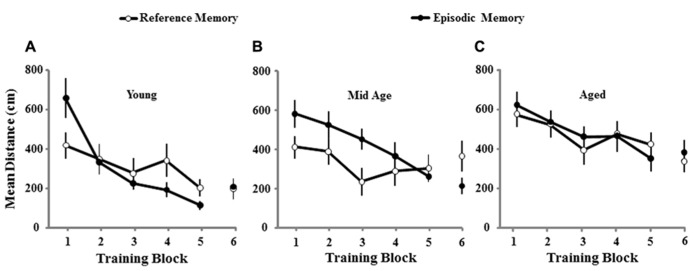
**Mean path length to find the escape platform during spatial discrimination training conducted 10 days following the initial episode of spatial training.** The mean distance traveled (±SEM) per training block in the spatial discrimination task for young **(A)**, middle-age **(B)**, and aged **(C)** rats trained for reference memory (open circles, *n* = 10/11/16, young/Mid Age/aged) or episodic memory (filled circles, *n* = 12/15/16, young/Mid Age/aged). Note that young and middle-age rats exhibit better performance on the first training block for the reference memory platform location, relative to episodic memory training to a new location, suggesting long-term spatial reference memory for these age groups.

A repeated measures ANOVA on the escape distances across the six training blocks for the second training session confirmed a main effect of age [*F*(2,74) = 9.06; *p* < 0.0005] and revealed an interaction of the training procedure, reference or episodic memory, and performance across trial blocks [*F*(5,370) = 3.21; *p* < 0.01]. *Post hoc* tests indicated that all age groups were different from each other with the shortest and longest distances observed for young and aged animals, respectively. Further examination for each training condition indicated an age effect [*F*(2,40) = 5.8; *p* < 0.01] for the cohort trained for episodic memory and *post hoc* tests indicated that, similar to the initial spatial training, young animals exhibited superior performance relative to aged and middle-age, which were not different from each other. In contrast, for reference memory training, when the platform was located in the same position, an age effect [*F*(2,34) = 3.99; *p* < 0.05] was observed due to poor performance of aged animals relative to the other two groups, which were not different from each other. The results suggest that aged and middle-age animals exhibit impaired episodic spatial memory; however, relative to aged animals, middle-age exhibit superior performance for reference memory, suggesting an age difference in the retention of reference spatial information across the 10-day interval. To determine age differences associated with the type of memory examined, the effect of training for reference and episodic memory was examined in each age group. The analysis indicated an interaction of the type of memory across training trials for middle-age [*F*(5,120) = 2.84; *p* < 0.05] and young animals [*F*(5,100) = 2.93; *p* < 0.05] due to better performance on the initial training blocks for the reference memory training, relative to training the animals to a novel platform location (**Figure 4**). The results confirm that young and middle-age rats exhibited long-term spatial reference memory, resulting in an initial decrease in path length for training to the same (i.e., reference) platform location relative to training to a new (i.e., episodic) platform location. Older animals did not exhibit this benefit, indicating impaired learning and or increased forgetting.

**FIGURE 5:**
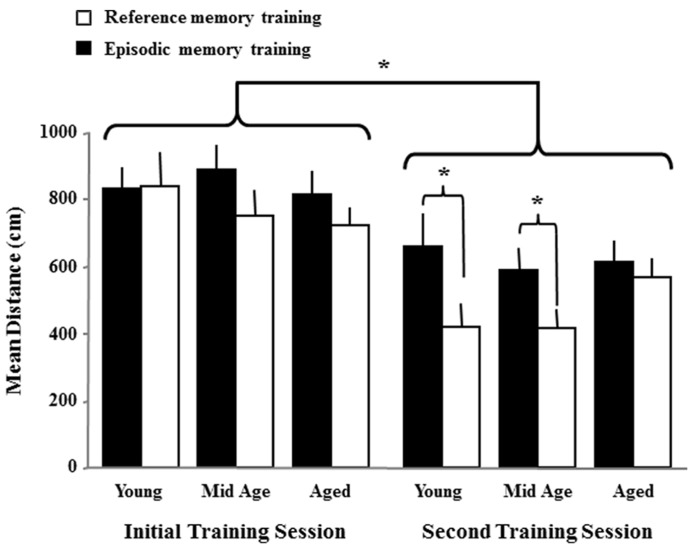
**Carryover effects due the initial spatial training.** The figure illustrates the mean distance + SEM to escape during the first training block for the initial and second spatial training sessions. The distance decreased across sessions, regardless of age indicating a carryover effect for some aspects of the task. An effect of the platform location was observed for the second session of training due to young and middle-age rats exhibiting reduced escape distance when the platform was in the same location as the initial training session (reference memory training, open bars) relative to a new platform location (episodic memory training, filled bars). Asterisks indicate a significant difference (*p* < 0.05) from the first to the second session across age and treatment groups and for location effects within each age group during the second session of training.

To determine if there were carryover effects associated with the initial training, a repeated measures ANOVA was performed on the first block of trials from the initial training session and the first block from the second spatial training session. The results indicate a decrease in path length [*F*(1,74) = 43.7, *p* < 0.0001] from the first to the second session and a main effect of the platform location [*F*(1,74) = 6.55, *p* < 0.05] in the absence of an age effect or an interaction (**Figure 5**). Examination of platform location and repeated measures effects within each age group indicated that each age group exhibited a reduced path length on the first block of the second training session (aged *p* < 0.01; middle-age *p* < 0.0005; young *p* < 0.001). One-tailed *t*-tests on the first block for the second training session were employed to determine whether the animals in each age group benefited from a reference memory for the platform remaining in the same location. The results indicated that only young and middle-age animals exhibited improved performance (reduced escape distance, *p* < 0.05) for escape to the same location relative to the novel platform position. These results indicate that all animals, regardless of age, exhibit better performance across days of training, likely due to carryover of procedural aspects of the task and that only young and middle-age animals benefited from a carryover of reference memory for the platform location.

**FIGURE 6:**
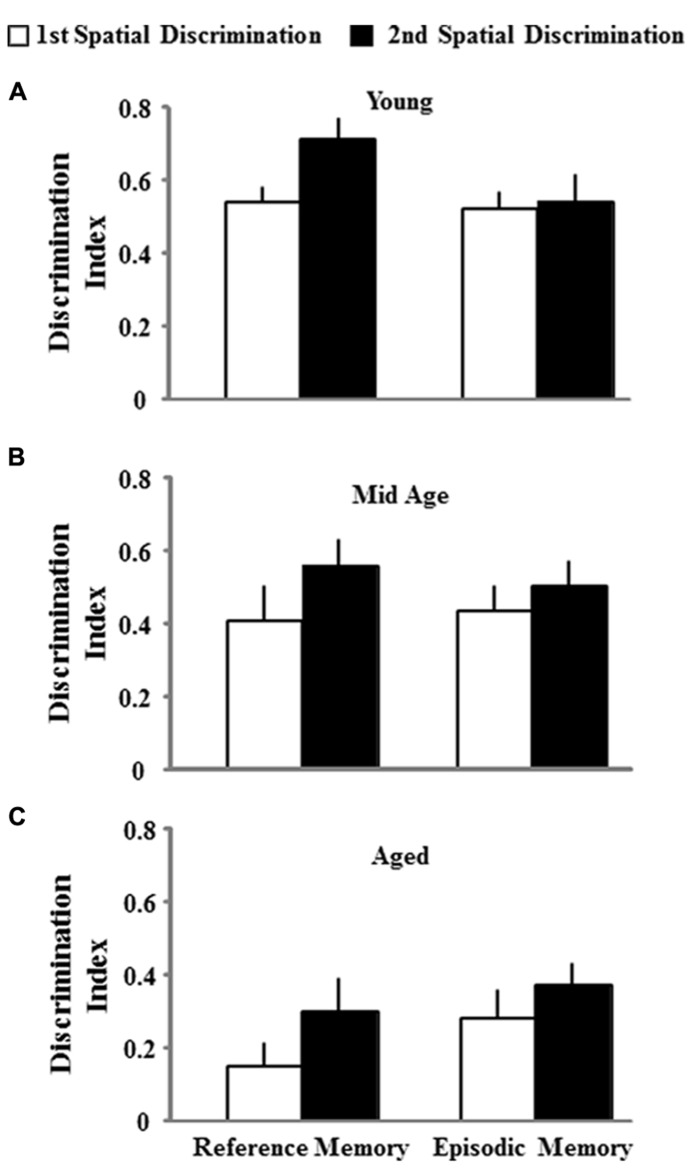
**Mean discrimination index scores (+SEM) for the acquisition probe trials for both, the first spatial discrimination task (open bar) and the second spatial discrimination task (filled bar), separated according to platform location for young **(A)**, middle-age (Mid Age) **(B)**, and aged **(C)** animals.** All discrimination index values were significantly different from chance performance (*p* < 0.05). Aged rats exhibited lower scores relative to the other two groups, regardless of platform location.

**FIGURE 7:**
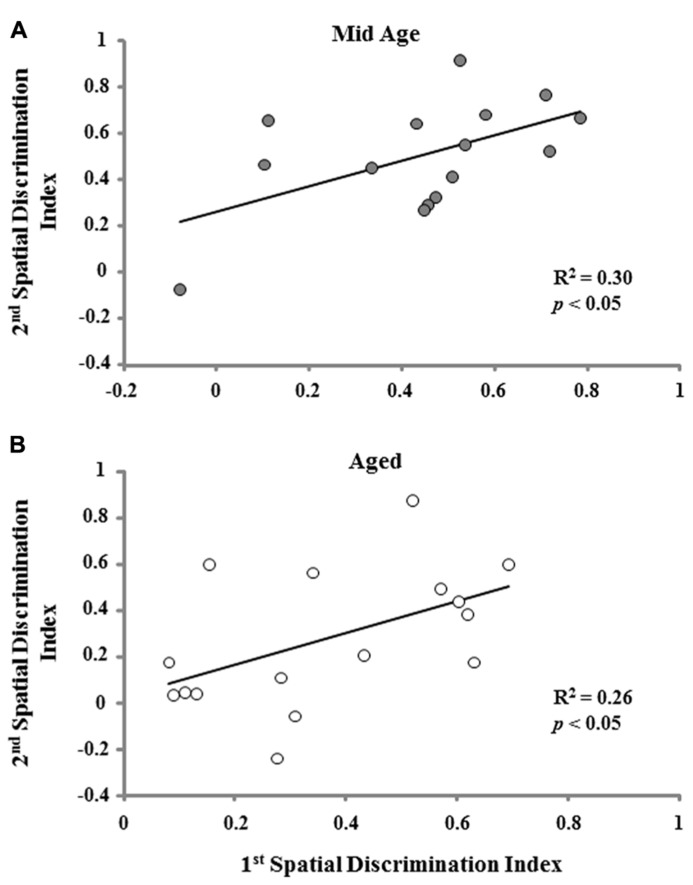
**A positive correlation was observed for the acquisition discrimination index scores from the initial spatial training session and episodic memory training to a novel platform position during the second training session for middle-age (Mid Age) (A) and aged **(B)** animals**.

One group *t*-tests on the discrimination index scores for the acquisition probe trial of the second training session indicated that each age and treatment group performed above chance. However, an ANOVA on the discrimination index scores from the second training session indicated an effect of age [*F*(2,74) = 9.022; *p* < 0.001] in the absence of an effect of the type of memory procedure employed (**Figure 6**). *Post hoc* tests indicated that aged rats demonstrate poorer learning performance (i.e., a lower discrimination index score) compared to both middle-age and young animals. To determine if the age-related impairment was reliable across the two training sessions, a regression analysis was conducted on the acquisition discrimination index scores across the two sessions within each age group. When the data for both the reference and episodic cohorts were combined, the results indicated that the performance on the first task could reliably predict future performance only in aged animals (*R*^2^ = 0.15, *p* < 0.05) and there was a tendency (*p* = 0.09) for middle-age animals. Subsequent analysis limited to each memory-training paradigm indicated that the discrimination index for the first training session could predict the discrimination index for episodic memory training to a novel location in middle-age (*R*^2^ = 0.30, *p* < 0.05) and aged (*R*^2^ = 0.26, *p* < 0.05) rats (**Figure 7**). The discrimination index for the first training session could not predict the discrimination index for reference memory training to the same location, examined 10 days later. Thus, for middle-age and aged animals, the ability to learn the escape location on the first spatial task could predict subsequent performance when animals were required to learn a new escape location indicating that the episodic memory task can detect a reliable deficit in spatial memory. Furthermore, impaired episodic memory is not a reliable predictor of reference memory performance, since an initial impairment in learning a spatial location did not predict performance for repeated testing to the same location. 

**FIGURE 8:**
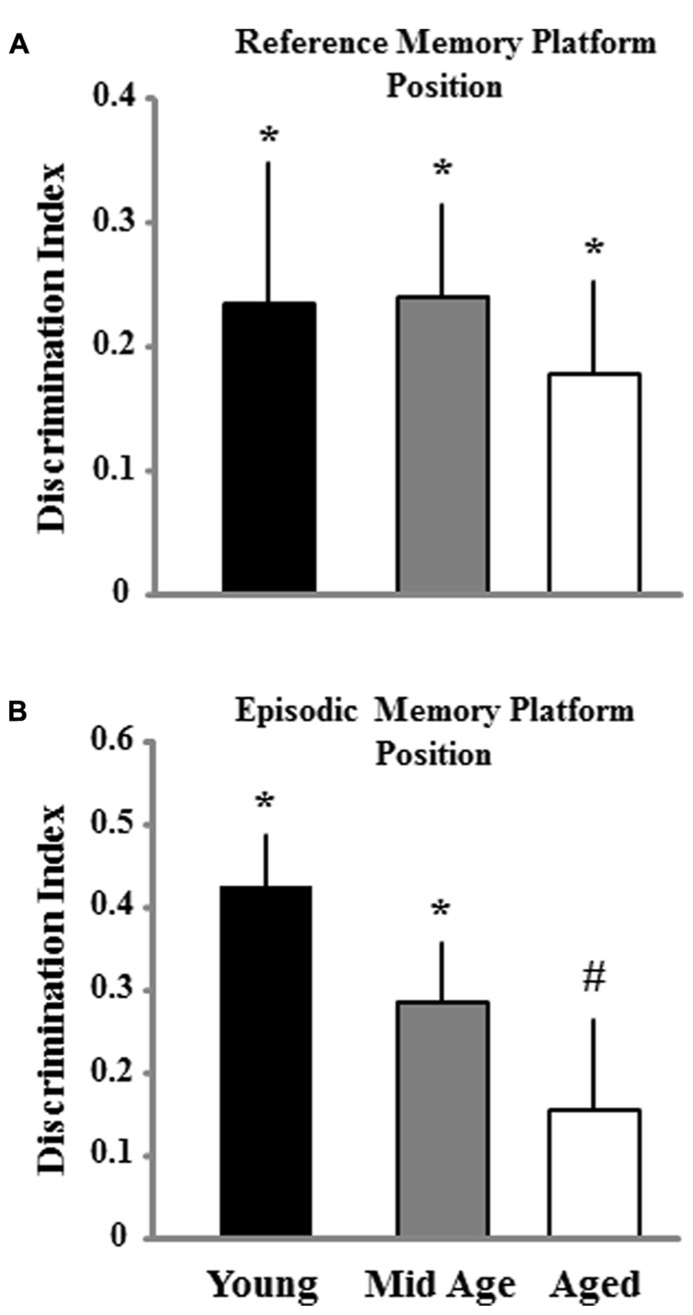
**Discrimination index for the retention probe trial for animals trained to either the reference (A) or episodic platform position **(B)** during the second session of spatial training.** The retention probe trial was instituted 24 h after the final block of training in order to assess spatial memory abilities for young, middle-age (Mid Age), and aged animals. Asterisk and pound sign signifies a significant difference from chance performance and young animals, respectively (*p* < 0.05).

Impaired episodic spatial memory involving long delays (6–24 h) emerges in middle-age (Foster, 2012a; Foster et al., 2012). A second probe trial to examine retention was conducted 24 h after the final training block (**Figure 8**). For animals trained on the episodic memory task, an ANOVA indicated a tendency (*p* = 0.12) for an age difference and *post hoc* tests confirmed the predicted difference between aged and young animals, while middle-aged animals were not different from either group. In the case of animals trained for reference memory, no age difference was observed. Finally, one group *t*-tests indicated that the discrimination index scores for the retention probe trial were above chance for all ages and for both conditions, except for aged animals in the episodic memory group. Again, the results indicate that aging is associated with impaired episodic memory and performance differences across ages are minimized for reference memory training.

### LONG-TERM REFERENCE MEMORY TRAINING

**FIGURE 9:**
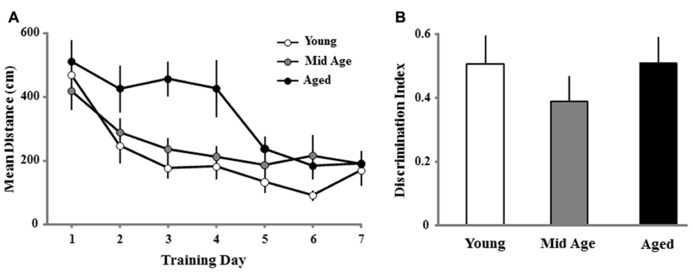
**For reference memory training across days, aged animals exhibit a slower rate of acquisition and no impairment in the maintenance of a reference memory.** **(A)** Mean path length to find the escape platform during the long-term reference memory spatial discrimination task in the water maze. Data represents the mean distance traveled (±SEM) per training day (four trials/day) for young (open circle, *n* = 14), middle-age (Mid Age, gray circle, *n* = 18), and aged (filled circle, *n* = 12) rats. Note that aged animals exhibit an initial impairment observed as a longer escape path length. **(B)** Discrimination index measure for a retention probe trial performed 24 h after the final day of reference memory training. Discrimination index values were all significantly different from chance (*p* < 0.05) and no age difference was observed for the reference memory.

Most studies that examine age-related changes in reference memory employ three to four trials per session and training is conducted over several consecutive days (Foster, 2012b). In the current study, young and middle-age animals exhibited a benefit from reference memory training observed during the first training block of the second training session (**Figure 5**). The difference in reference memory may be due to slower acquisition of aged animals such that they required more trials. Alternatively, the apparent lack of a reference memory for aged animals for the first block of the second session could have resulted from forgetting over the 10-day period between sessions. To examine this possibility, a subgroup of animals continued with reference memory training 24 h following the last probe trial of the initial spatial discrimination task (**Figure 1**). The methods remained the same with the exception that following the first day of training, only one block (four trials/day) of training was performed per day for seven consecutive days. **Figure 9A** shows the mean escape path length per day, over the course of 7 days, for each age group (young, *n* = 14; middle-age, *n* = 18; and aged, *n* = 12). A repeated measures ANOVA indicated a training effect [*F*(6,246) = 16.828; *p* < 0.0001] as well as an effect of age [*F*(2,41) = 6.882; *p* < 0.005] and a tendency (*p* = 0.055) for an interaction. *Post hoc* analysis indicated that aged animals exhibited longer path lengths relative to young and middle-age animals and this difference could be observed early in training, on days 3 and 4. Repeated measures ANOVAs for each age group indicated that all groups exhibited learning over the course of training. Twenty-four hours after the seventh and final day of spatial training, a reference memory probe trial was instituted (**Figure 9B**). One group *t*-tests indicated that all groups exhibited performance above chance and an ANOVA indicated no age difference in the discrimination index. The results indicate that the oldest animals exhibited impairments for the initial acquisition of the spatial reference memory. Nevertheless, aged animals can acquire a reference memory with continued training.

**FIGURE 10:**
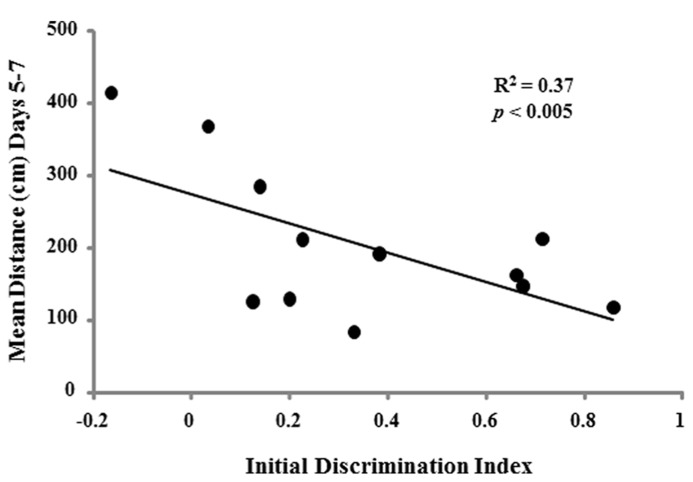
**Acquisition discrimination index scores for the initial day of spatial training is related to acquisition of a spatial reference memory in aged animals. ** For aged rats (*n* = 12), a significant correlation was observed for the initial acquisition discrimination index scores and the mean escape distance for days 5–7 on the reference memory task.

To determine whether the ability to learn a spatial location following the single day of massed training could predict acquisition performance when animals were trained across consecutive days, regression analysis was performed for each age group using the discrimination index of the first spatial discrimination task and the averaged escape distances for days 2–4, days 5–7, and the discrimination index for the reference memory task. A negative correlation was observed only in the aged cohort for the acquisition discrimination index and the averaged escape distance for days 5–7 (*R*^2^ = 0.37, *p* < 0.05; **Figure 10**).

## DISCUSSION

The water maze is the gold standard for examination of hippocampal function and a primary model for the characterization of age-related cognitive impairments. One unique feature of the swim task is that it is amendable to multiple testing sessions in order to examine the reliability of deficits, as well as the success of treatments. In this regard, the utility of the task depends on a number of factors including the training procedures. In the current study, we observed that young, middle-age, and aged rats exhibited carryover effects (i.e., memory) from prior testing; however, the extent of carryover influences varied by age.

### PROCEDURAL MEMORY IS INTACT DURING AGING

All groups, regardless of age, exhibit better performance for the second training session 10 days following the first training session indicating memory for certain aspects of the task. These carryover effects likely include memory for the procedural aspects of the task, memory for the spatial cues that remain constant across sessions (i.e., reference memory), and the use of a spatial mapping strategy. When reference memory training is conducted in two different environments, all age groups exhibited improved acquisition in the second environment; nevertheless, aged animals initially characterized as impaired for acquisition of the first environment reliably performed below age-matched animals initially characterized as unimpaired (Colombo and Gallagher, 2002). The results suggest that a subgroup of the oldest animals exhibit a reliable deficit in acquiring new spatial information. In contrast, longitudinal studies demonstrate that spatial training on the water maze during young adulthood or middle-age facilitates the use of a spatial mapping strategy later in life, which provides advantages for reference memory when tested during aging (van Groen et al., 2002; Hansalik et al., 2006). For longitudinal testing, impaired acquisition of a new reference memory manifest only in very old animals (~24 months), observed as the absence of improvement in performance between the first trial and subsequent training trials (Kikusui et al., 1999). However, impairments were also evident for the first trial suggesting that sensory-motor deficits may mediate impaired acquisition of a reference memory in the oldest animals. Indeed, aging of cortical regions linked to spatial perception memory (Chiba et al., 2002), skill or source information for the task procedures (Winocur and Moscovitch, 1990), as well as subcortical neuromodulatory systems (Foster, 2012b) likely contribute to deficits in reference memory performance observed for the oldest animals.

### DEFICITS FOR EPISODIC SPATIAL MEMORY EMERGE IN MIDDLE-AGE

Repeated training, involving the acquisition and retention of episodic spatial information, is sensitive to the emergence of impairments in middle-age (Foster, 2012a; Foster et al., 2012). For both longitudinal and cross-sectional studies, no age difference is observed for the first (i.e., information) trial. However, age differences begin to emerge in middle-age either as impaired performance on subsequent training trials or as a retention deficit observed for long (<1 h) delays (Kikusui et al., 1999; Driscoll et al., 2006; Bizon et al., 2009; Kumar and Foster, 2013). In the current study, a subset of middle-age and aged animals exhibited a learning impairment observed as a greater escape distance and decreased acquisition probe trial discrimination index for the initial spatial task. While the proportion of aged impaired animals may differ across studies, due to the criteria employed, it is important to note that the propensity for impaired acquisition of episodic spatial memory increased from middle-age (~25%) to aged (~60%) animals. This deficit was also observed for some aged and middle-aged animals during the second session of episodic memory training to a novel escape location. Indeed, in the episodic memory cohort, the discrimination index scores for aged and middle-age animals were correlated across the two training sessions. Thus, impaired acquisition of episodic information emerges in middle-age, and while performance is variable across animals, performance is reliable across training sessions. Memory deficits were also more prominent with advancing age. In contrast to young and middle-age animals, aged animals in the reference memory cohort did not exhibit a benefit of training to the same platform location during the second episode of spatial training. Furthermore, impaired memory 24 h after the second episode of spatial training was prominent for aged animals trained in the episodic memory condition, with the performance of middle-age animals intermediate between young and aged. The results are consistent with the idea that delay-dependent retention of episodic spatial memories exhibits a progressive decline during aging (Foster, 2012a; Foster et al., 2012).

### AGING AND ACQUISITION OF A SPATIAL REFERENCE MEMORY

No age difference was observed for the discrimination index scores for acquisition or retention following two sessions of training to the same spatial location (i.e., reference memory). Thus, despite a deficit for processing episodic spatial information with repeated training, aged animals can acquire a spatial reference memory. In this case, impaired episodic memory may contribute to the rate at which a reference memory is acquired. For example, during the second spatial training session, aged animals did not show an initial benefit of having been trained to the reference location. Similarly, when reference memory training was presented over several days, impairment in the rate of acquisition was limited to the initial days of training in aged animals. Again, with additional training, aged animals improved to match the performance of the younger groups and were able to establish a reference memory that extended over the 24 h period. Finally, we observed a relationship between the acquisition in the initial spatial training task and learning a reference memory following several days of training, suggesting that episodic memory may contribute to spatial reference memory.

Together, the results of the current study point to a reliable and progressive impairment in episodic spatial memory during aging. Reference memory impairments were limited to the rate of acquisition for the oldest animals. The results suggest that tests of episodic spatial memory are more sensitive for detecting the emergence of cognitive decline. A relationship was observed between the decline in episodic and reference memory, suggesting that impaired episodic memory may be a prerequisite for more severe impairments including the inability to acquire reference memories. Alternatively, differences in the senescence of the mechanisms that underlie the two forms of memory may underlie the discrepancy in the onset of impairments.

## Conflict of Interest Statement

The authors declare that the research was conducted in the absence of any commercial or financial relationships that could be construed as a potential conflict of interest.
